# Structural and in vitro anticancer properties of the kaempferol–lactoferrin complex

**DOI:** 10.1002/fsn3.4479

**Published:** 2024-09-19

**Authors:** Peiyu Xue, Hongmei Zhao, Xinyong You, Weiming Yue

**Affiliations:** ^1^ School of Biology and Food Engineering Anyang Institute of Technology Anyang China; ^2^ Department of Thoracic Surgery Qilu Hospital of Shandong University Jinan China

**Keywords:** anticancer activity, complexation mechanism, kaempferol, lactoferrin, synergistic effect

## Abstract

Lactoferrin and polyphenols are common natural functional compounds. Their interactions and the consequential alterations in functional activity have received widespread attention. The work aimed to investigate the interaction between lactoferrin and kaempferol, as well as evaluate the in vitro anticancer properties of the lactoferrin–kaempferol complex. The results of the spectra experiments revealed that lactoferrin and kaempferol are capable of forming complexes to quench the endogenous fluorescence of lactoferrin. Further insight into the binding mechanism was gained through molecular docking and molecular dynamics simulations. These analyses suggest that both hydrophobic interactions and hydrogen bonding are essential factors in the interaction between lactoferrin and kaempferol. Furthermore, the MTT assay and apoptosis by flow cytometry were conducted, revealing a synergistic effect of kaempferol and lactoferrin on the inhibition of HeLa cell proliferation. The findings from this investigation could improve our understanding of lactoferrin's interaction with polyphenols and the role of the lactoferrin–kaempferol complex in inhibiting cancer cell proliferation.

## Graphical abstract

Lactoferrin and kaempferol could form complexes through hydrogen bonding and hydrophobic interactions and reveal a synergistic effect on the inhibition of HeLa cell proliferation.

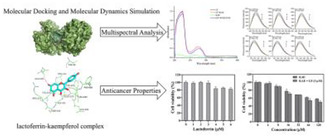

## 1 INTRODUCTION

Lactoferrin (LF) is a multifunctional protein that is found in dairy products and plays a crucial role in various biological processes. It is known for its ability to bind and transport iron, contributing to iron metabolism and the synthesis of hemoglobin (Legrand, 2016; Tsukahara et al., 2020). In addition, various biological functions of LF have been documented, encompassing its ability to combat cancer, bacteria, viruses, fungi, inflammation, and regulate the immune system (Tran et al., 2023). Moreover, recent studies have investigated and implemented LF‐based carriers for the transportation of bioactive substances, encompassing complexes, emulsions, and nanoparticle components (Liu et al., 2018). These findings suggest that LF holds promise as an innovative resource in the realm of biomaterials advancement.

Kaempferol (KAE) is a naturally occurring flavonoid found in various plants, such as strawberries, Ginkgo biloba leaves, tea, grapes, and quinoa (Qian et al., 2023; Zhou & Li, 2020). KAE has gained significant attention due to its potential health benefits. Numerous preclinical studies have demonstrated that KAE exhibits a wide range of biological activities, encompassing anticancer, anti‐inflammatory, antimicrobial, antioxidant, cardiovascular, and neuronal protection, as well as antidiabetic effects (Zheng et al., 2022). Established studies reveal that the mechanism of KAE relies on inhibiting the proliferation of various cancer cells through cell cycle arrest or inducing apoptosis (Imran et al., 2019). KAE could achieve cell cycle arrest by antagonizing estrogen‐related receptor α (ERRα) activity (Wu et al., 2017), inhibiting epidermal growth factor receptor (EGFR) and estrogen activity (Kim et al., 2016; Yao et al., 2016). In addition, many studies have been dedicated to investigating the interaction between KAE and various proteins, such as human serum albumin, bovine serum albumin, and α‐lactalbumin, to evaluate the functional activity of protein‐KAE complexes or the possibility of proteins serving as KAE delivery carriers (Diao et al., 2021; Mohammadi & Moeeni, 2015).

Although there have been many reports on the interaction between KAE and various proteins, research investigating the impact of LF on the anticancer activity of KAE is currently limited. The objective of this study was to investigate the interaction between LF and KAE through the utilization of spectroscopic methods. Furthermore, molecular docking was employed to assess the binding modes of LF and KAE, while molecular dynamics simulations were utilized to determine their respective stabilities. Moreover, the cytotoxicity and induction of cell apoptosis resulting from the LF–KAE complex on HeLa cells were assessed through MTT assays and flow cytometry. This investigation could enhance our understanding of the interaction between LF and polyphenols, as well as the impact of the LF–KAE complex on cellular viability.

## 2 MATERIALS AND METHODS

### 2.1 Materials

Bovine LF with 95% purity and KAE were purchased from Shanghai YuanYe Biological Technology Company (Shanghai, China). 3‐(4,5‐Dimethylthiazol‐2‐yl)‐2,5‐diphenyltetrazolium bromide (MTT) was obtained from Sigma‐Aldrich. The mixed solution containing penicillin–streptomycin, Dulbecco's modified Eagle medium (DMEM), trypsin, and dimethyl sulfoxide (DMSO) was procured from Thermo Fisher Scientific. Fetal bovine serum (FBS) was sourced from Clark Bio. Annexin V‐FITC/PI Apoptosis Detection Kit was purchased from Simubiotech. Ethanol (spectroscopy grade) was purchased from Aladdin Biological Co., Ltd. The rest of the reagents were of analytical grade.

### 2.2 Ultraviolet (UV) spectra measurement

Lactoferrin (1.0 × 10^−5^ mol L^−1^) was prepared with phosphate buffer solution (PBS, 10 mM pH 7.4). KAE was prepared as a 0.5 mM stock solution through the dissolution of an appropriate quantity of the compounds in ethanol, followed by dilution with PBS to achieve the desired concentrations. The ethanol volume fraction in final mixtures was below 2% (Huang et al., 2020).

The UV spectroscopic measurement of LF was performed on a UV–Vis Spectrophotometer (Persee T9CS) under two conditions: in the absence and presence of KAE. The measurements were conducted in the wavelength range of 200–400 nm (Pattanayak et al., 2017). Spectra of LF and KAE were obtained at a fixed concentration of 5.0 × 10^−6^ mol L^−1^.

### 2.3 Fluorescence spectrum measurements for steady state and synchronous fluorescence

The fluorescence spectra of LF in the presence and absence of KAE were analyzed using an F‐7000 FL Spectrophotometer (Hitachi Company). The excitation and emission slit widths were both configured to 2.5 nm, while the scan speed was set at 240 nm/min. The excitation light wavelength was set to 280 and 295 nm. LF was maintained at a concentration of 5.0 × 10^−6^ mol L^−1^, while the concentration of KAE was systematically varied from 0.0 to 10 × 10^−6^ mol L^−1^ in increments of 2 × 10^−6^ mol L^−1^. The mixed solution was subjected to a water bath cauldron for a duration of 60 minutes at temperatures of 298.15, 304.15, and 310.15 K before fluorescence measurements were taken (Dai et al., 2022). The following equation (Equation 1) was used to account for fluorescence intensities caused by inner‐filter effects in this study (van de Weert & Stella, 2011).

(1) $$F_{\text{corr}}=F_{\mathrm{obs}}\times e^{\left(A_{\mathrm{ex}}+A_{\mathrm{em}}\right)/2}$$

$F_{\text{corr}}$ represents the corrected fluorescence intensity, while $F_{\mathrm{obs}}$ denotes the background‐subtracted fluorescence intensity of the sample. The absorbance values at excitation and emission wavelengths are indicated as $A_{\mathrm{ex}}$ and $A_{\mathrm{em}}$, respectively.

In addition, the Stern–Volmer equation (Equation 2) was used to further analyze the fluorescence data to determine the mechanism of fluorescence quenching in the case of KAE and LF (Djeujo et al., 2022).

(2) $$F_{0}/F=1+K_{\mathrm{SV}}\left[Q\right]=1+K_{q}\tau_{0}\left[Q\right]$$

*F*_0_ and *F* represent the fluorescence intensities without and with KAE, respectively. [*Q*] stands for the concentration of KAE. Additionally, *K*_sv_ denotes the Stern‐Volmer quenching constant, *K*_q_ signifies the biomolecule quenching rate constant, and τ_0_ refers to the average fluorescence lifetime of LF in the absence of a quencher, with a magnitude of approximately 10^−8^ s (Wang et al., 2021).

Meanwhile, a double‐logarithmic equation (Equation 3) was used to ascertain the binding constant (*K*_a_) and the number of binding sites (*n*) (Perusko et al., 2017).

(3) $$\log\frac{F_{0}-F}{F}=\log K_{a}+n\log\left\lfloor Q\right\rfloor$$

Synchronous fluorescence spectra were obtained with a wavelength interval (Δλ) of 15 nm for tyrosine residues and 60 nm for tryptophan residues (Jing et al., 2021). The experimental setup was consistent with that of the static fluorescence testing system. Subsequent calculations were conducted to determine the corresponding ratios of synchronous fluorescence quenching, denoted as RSFQ [RSFQ (%) = 1 − *F*/*F*_0_].

### 2.4 Molecular docking between LF and KAE

The three‐dimensional structure of diferric bovine LF (1BLF) was obtained from Protein Data Bank (Moore et al., 1997). All the co‐crystallized oligosaccharides as well as small molecules, including 2‐acetamido‐2‐deoxy‐β‐d‐glucopyranose, carbonate ions, Fe (III) ions, and water molecules, were removed by Chimera 1.11. Afterward, the structure of LF was prepared by AutoDockTools‐1.5.6. GaussView and Gaussian 09 W were used to develop and optimize the structure of KAE. Molecular docking between LF and KAE was performed with a grid box of 90 × 90 × 90 points by AutoDockTools‐1.5.6. The center values of the grid box were adjusted as for *x* = 50.203, *y* = 60.125, and *z* = 22.807, respectively. The docking conformation of KAE with the lowest binding energy was visualized by PyMOL.

### 2.5 Molecular dynamics simulation of the LF–KAE complex

Molecular dynamics simulations were conducted using the LF–KAE complex with the lowest binding energy. The topology of KAE was generated using the CGenFF server, while the topology of LF was created using the CHARMM36 all‐atom force field. The LF–KAE complex was solvated in a cubic water box, and 13 chloride ions were randomly added to achieve charge neutrality. This study conducted equilibrations for 0.1 ns under constant number of particles pressure–temperature and constant number of particles volume–temperature conditions, respectively. The position restraints were then released in order to conduct a 70 ns molecular dynamics simulation using GROMACS 2019, with the entire system being maintained at the desired pressure and temperature. Subsequently, the root mean squared deviation (RMSD) values were computed for both KAE and the LF backbone.

### 2.6 Cell culture and MTT assay

HeLa cells, originally obtained from the Cell Bank of Chinese Academy of Science, were stored in a −80°C freezer. To initiate cell culture, the frozen cells were thawed and recovered by immersing them in a 37°C water bath. Following thawing, the cells were cultured in DMEM supplemented with 10% inactivated FBS and then incubated at a constant temperature of 37°C under a 5% CO_2_ and 95% air atmosphere with consistent humidity. The culture medium was refreshed every 2 days to maintain proper nourishment.

In the MTT assay, HeLa cells were seeded in 96‐well plates at a concentration of 4 × 10^4^ cells per well. On the following day, different compounds were added to the wells at specified concentrations according to the experimental design. Specifically, the treatment concentrations of KAE ranged from 0 to 128 μM in a series of dilutions, while the treatment concentrations of LF ranged from 0 to 6 μM. In the combination group, a fixed concentration of 3 μM LF was used. After the addition of compounds and a 24‐h incubation period, each well received 100 μL of fresh culture medium containing MTT. The cells were subsequently incubated for an additional 4 h, after which the culture medium was removed. Following this, 110 μL of DMSO was added to each well, and the plates were placed on a low‐speed shaker for 10 min to facilitate the complete dissolution of formazan crystals (Alizadeh Behbahani et al., 2020). Finally, the absorbance values at 490 nm were measured using a microplate reader (Synergy HT, BioTek) to determine changes in IC_50_ values as an indicator of cytotoxic effects.

### 2.7 Cell apoptosis analysis

According to the optimal concentration obtained from the MTT assay, cell apoptosis was detected using flow cytometry after incubation with KAE, LF, or LF–KAE complex. Briefly, following a 24‐h treatment, HeLa cells were digested using EDTA‐free trypsin and subsequently harvested. The cells were then washed twice with ice‐cold PBS. The binding buffer in the kit was used to re‐suspend the cells and adjust the cell concentration to 1 × 10^6^ cells per mL. Subsequently, 100 μL of cell resuspension was transferred into the sample tube and mixed with 5 μL of Annexin V‐FITC and then incubated away from light for 10 min before 5 μL PI was added. After mixing, the sample was further incubated in the dark for 5 min. Finally, 400 μL of binding buffer was introduced, and the sample was subjected to analysis via flow cytometry (BD Accuri C6).

### 2.8 Statistical analysis

Curve fitting and processing of statistical data were performed using the OriginPro 2021 software (OriginLab Corp.). IC_50_ values were obtained using GraphPad Prism 8.0.

## 3 RESULTS AND DISCUSSION

### 3.1 UV spectroscopy analysis of LF–KAE system

**FIGURE 1:**
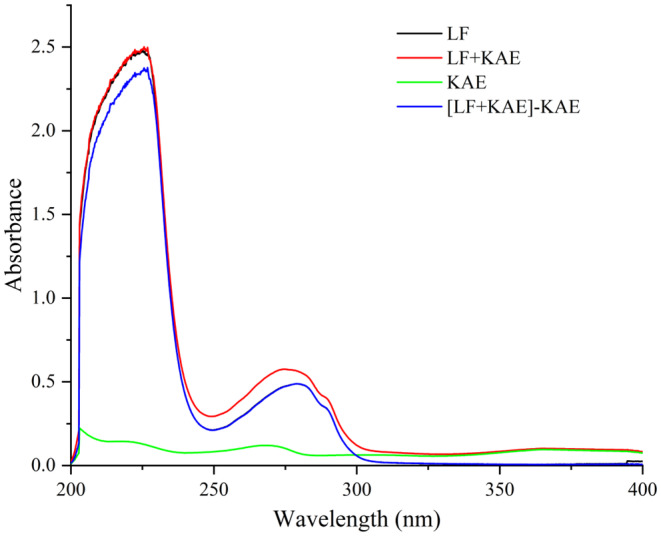
UV–visible absorption spectra of lactoferrin in the absence and presence of kaempferol. c (lactoferrin) = c (kaempferol) = 5.0 × 10^−6^ mol L^−1^. The absorption spectrum of [LF + KAE]‐KAE is obtained by subtracting the corresponding spectrum of KAE from that of the LF + KAE complex.

The UV spectroscopy technique is commonly employed to investigate the interactions between biological macromolecules and small molecules in academic research (Narva et al., 2016; Xue et al., 2022). Generally, a given protein has unique spectral absorption characteristics. It is expected that when small molecules or ligands interact with proteins, their UV–visible absorption profiles will change as a result of their complex formation (Ali & Al‐Lohedan, 2022). Figure 1 shows the results of the ultraviolet absorption spectrum. It is worth noting the spectral differences within the range of 220–228 nm. In this band range, KAE has a certain absorbance, and the absorbance value is in the range of 0.1–0.15. However, the absorption curve of the LF–KAE system almost coincides with that of LF within this band range. This phenomenon means that the addition of KAE affects the absorbance characteristics of LF. Furthermore, the disparity in absorption spectra between LF and [LF + KAE]‐KAE within the 220–228 nm further signifies this phenomenon. Similarly, Huang et al. investigated the interaction between LF and the flavonoids dihydromyricetin and myricetin using ultraviolet spectroscopy, and our findings were consistent with their results (Huang et al., 2020). Differences observed in the UV spectrum indicated potential interaction between LF and KAE.

### 3.2 Fluorescence quenching mechanism, binding constant, and number of binding sites of the LF–KAE system

**FIGURE 2:**
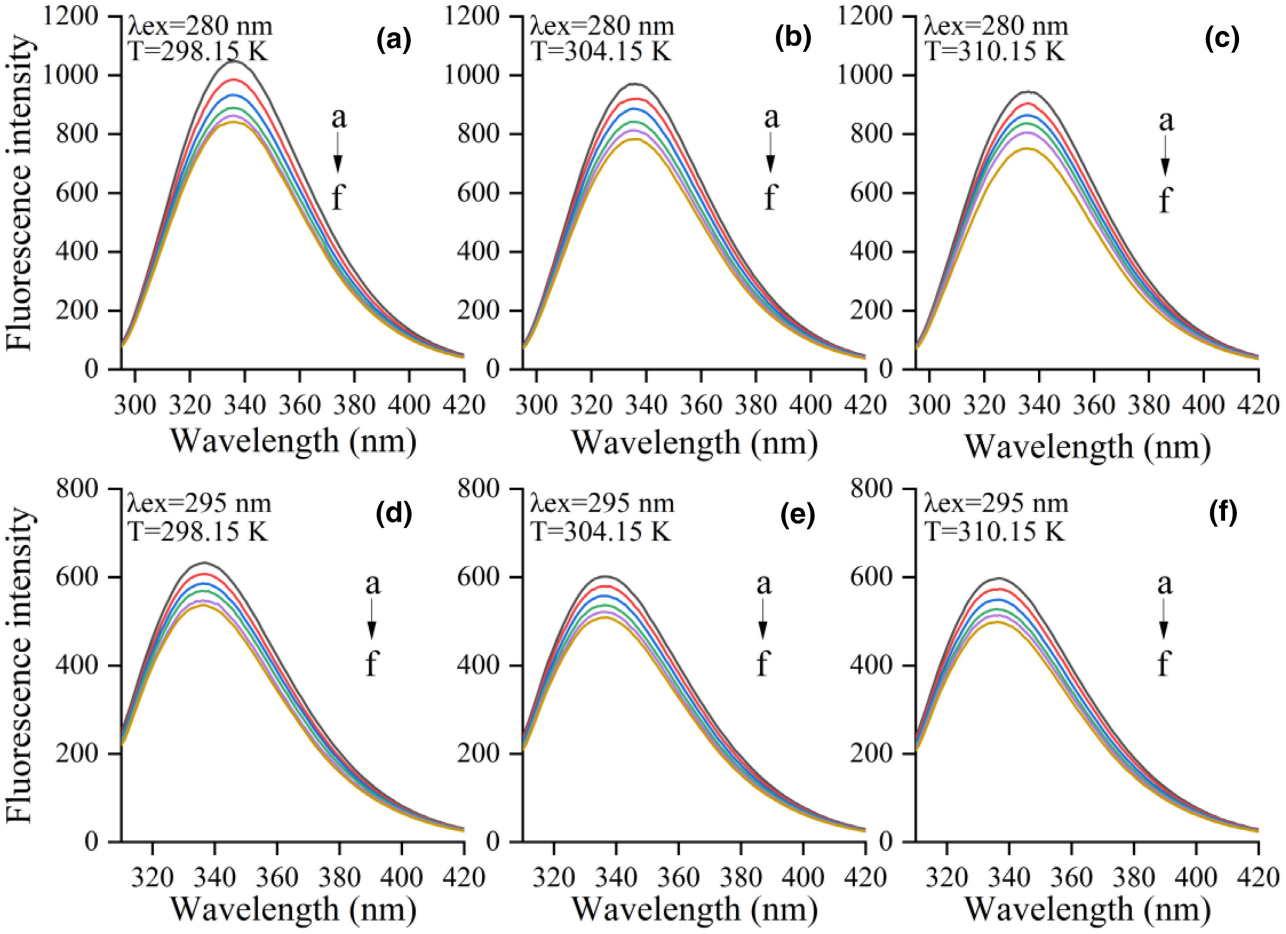
The fluorescence emission spectra of the LF–KAE system at excitation λ = 280 and 295 nm under 298.15, 304.15, and 310.15 K. The lactoferrin concentration was 5.0 × 10^−6^ mol L^−1^, while the kaempferol concentration was increased from 0 to 10 × 10^−6^ mol L^−1^ at an increment of 2.0 × 10^−6^ mol L^−1^ (a → f).

The aromatic amino acids in proteins, such as tryptophan, tyrosine, and phenylalanine can produce fluorescence under excitation (Kang et al., 2022). Changes in the fluorophore surrounding environments could significantly affect its intrinsic fluorescence intensity (Zhang et al., 2020). Fluorescence emission spectrum of LF–KAE system after excitation at 280 nm and 295 nm are plotted in Figure 2. As the concentration of KAE gradually increased, the fluorescence intensity of the system continuously decreased. This phenomenon indicates that KAE could quench the endogenous fluorescence of LF. Similar results were observed when KAE formed complexes with α‐lactalbumin and human serum albumin (Diao et al., 2021; Matei & Hillebrand, 2010).

**FIGURE 3:**
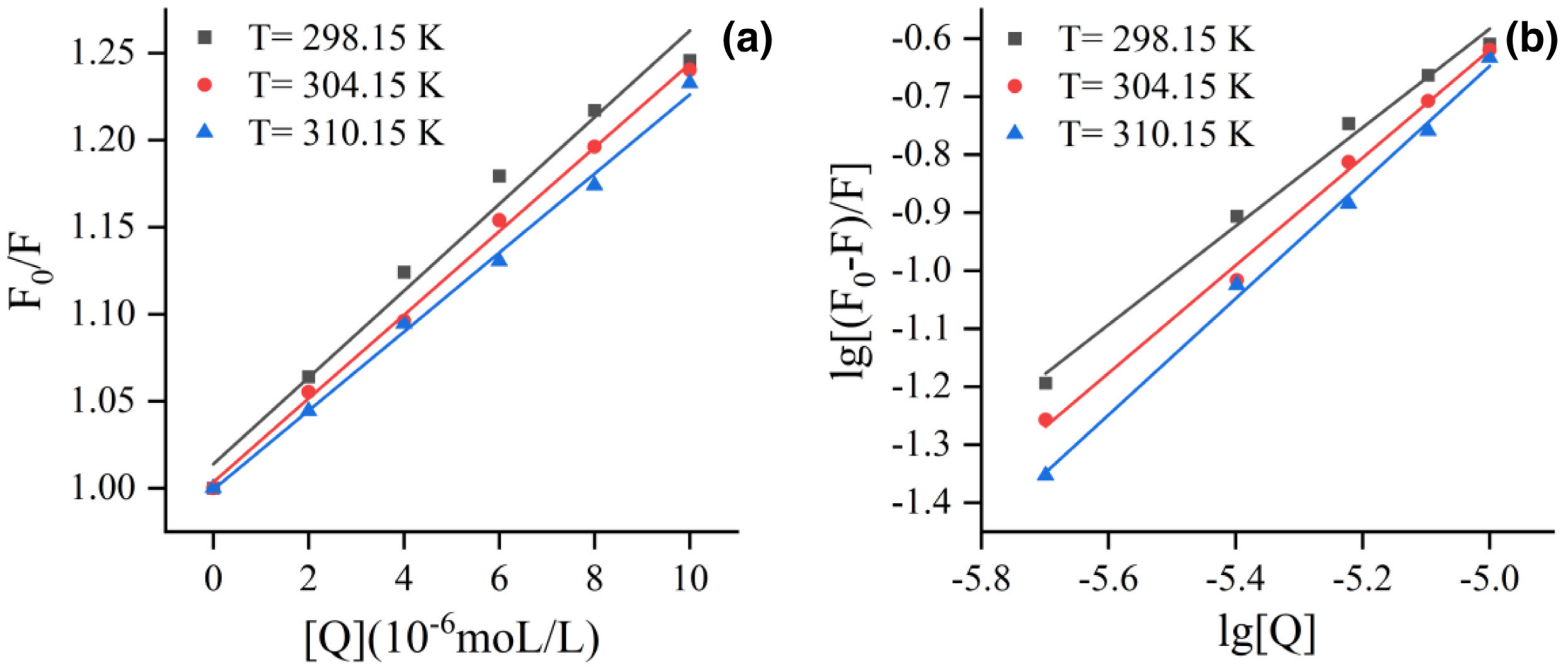
Stern–Volmer plot (a) and double logarithmic plot (b) for quenching of lactoferrin by kaempferol.

Dynamic quenching and static quenching represent the primary mechanisms by which ligand‐induced protein fluorescence quenching occurs (Xu et al., 2019). The Stern‐Volmer plots were depicted in Figure 3a, while the corresponding values of *K*_sv_ and *K*_q_ (where *K*_q_ is defined as *K*_sv_ divided by τ_0_) were documented in Table 1. The *K*_q_ is generally near 2.0 × 10^10^ L mol^−1^ s^−1^ in dynamic quenching processes (Li et al., 2018). As illustrated in Table 1, the calculated *K*_q_ values for LF–KAE system is two orders of magnitude higher than 2.0 × 10^10^ L mol^−1^ s^−1^, indicating the presence of static quenching during the binding process. Chen et al. (2016) believed that saikosaponin C could form a complex with human serum albumin, based on the fact that the quenching rate constant of the two was higher than the criterion of 2.0 × 10^10^ L mol^−1^ s^−1^. Furthermore, it can be observed that the Stern‐Volmer quenching constant *K*_sv_ exhibits an inverse relationship with temperature, which also indicated that the quenching mechanism of LF–KAE interaction is initiated through the formation of complexes (Feroz et al., 2012).

**TABLE 1:** The Stern–Volmer quenching constants and binding parameters of the lactoferrin–kaempferol system.

*T* (K)	*K*_sv_ (L mol^−1^)	*K*_*q*_ (L mol^−1^ s^−1^)	*K*_*a*_ (L mol^−1^)	*n*
298.15	2.49 × 10^4^	2.49 × 10^12^	4.60 × 10^3^	0.85
304.15	2.40 × 10^4^	2.40 × 10^12^	1.05 × 10^4^	0.93
310.15	2.27 × 10^4^	2.27 × 10^12^	2.31 × 10^4^	1.00

The binding constant (*K*_a_) and number of binding sites (*n*) in static quenching could be calculated based on fluorescence intensity data (Naik et al., 2014). The results depicted in Figure 3b and summarized in Table 1 reveal that the *K*_a_ values were determined to be at a magnitude of 10^4^ L mol^−1^, signifying a strong affinity between KAE and LF (Chen et al., 2016). The n values fell within the range of 0.8–1.0, indicating a near‐equivalent stoichiometry of 1:1 between KAE and LF. Moreover, elevating the temperature of the interaction resulted in an augmentation of both K_a_ and n values, which substantiates the involvement of hydrophobic interactions in the binding process of LF and KAE (Chen et al., 2003; Phopin et al., 2019).

### 3.3 Synchronous fluorescence spectra

**FIGURE 4:**
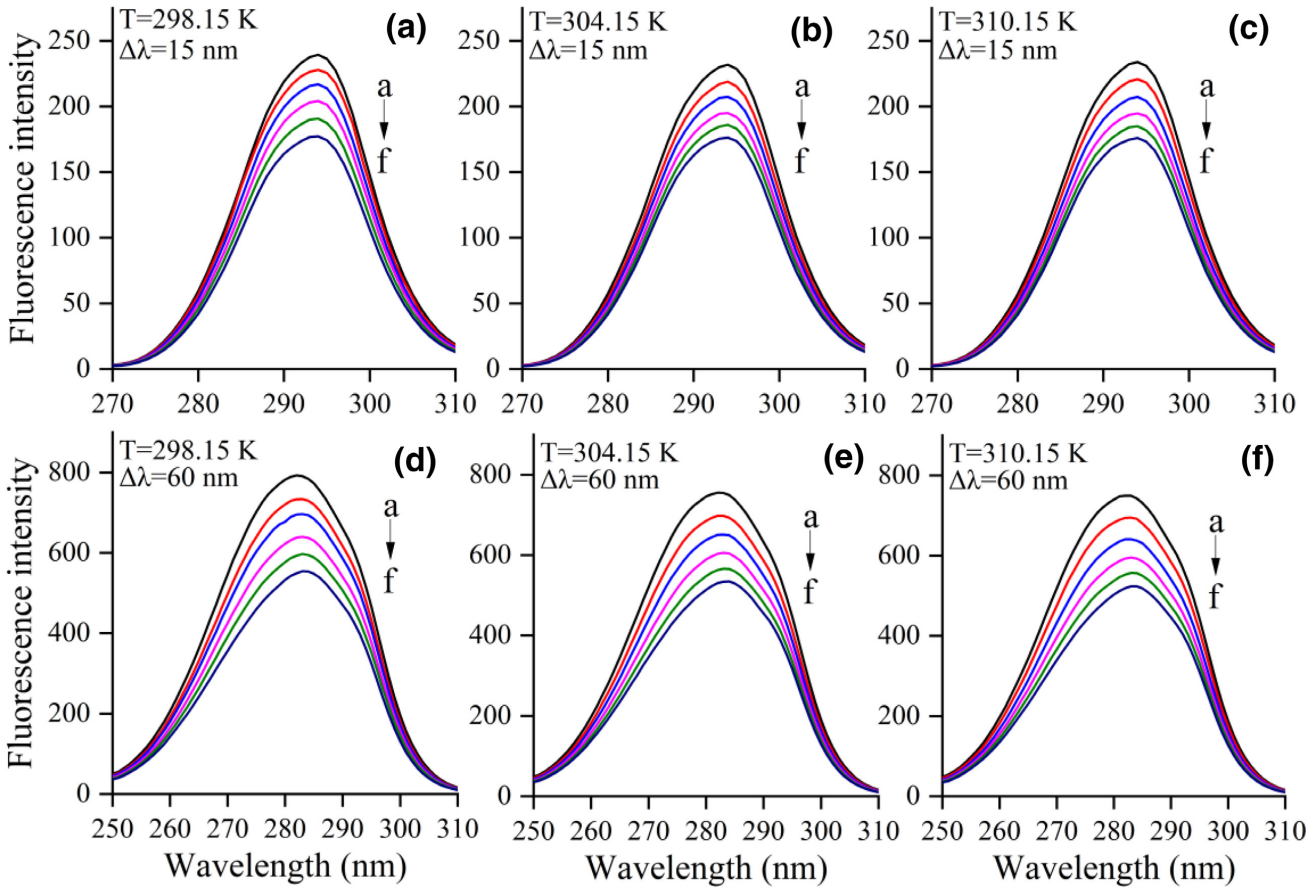
Synchronous fluorescence spectra of lactoferrin in the absence and presence of kaempferol at Δλ = 15 and Δλ = 60 nm. The lactoferrin concentration was 5.0 × 10^−6^ mol L^−1^, while the kaempferol concentration was increased from 0 to 10 × 10^−6^ mol L^−1^ at an increment of 2.0 × 10^−6^ mol L^−1^ (a → f).

Synchronous fluorescence spectroscopy is utilized to assess changes associated with the molecular environment surrounding chromophore molecules, specifically Tyr and Trp, by employing wavelength intervals (λ_em_–λ_ex_) of 15 and 60 nm, respectively (Li et al., 2022; Rahman et al., 2019). Figure 4 illustrates the synchronous fluorescence of LF in the presence or absence of varying concentrations of KAE. Upon gradual addition of KAE, there was a consistent decrease in the synchronous fluorescence intensity of LF and a noticeable red‐shift in the maximum emission wavelength of Trp residues (Figure 4d–f), while the maximum emission wavelength of Tyr residues (Figure 4a–c) remained unchanged. These results indicate that KAE increased the polarity of the microenvironment surrounding Trp residues, while the microenvironment surrounding Tyr residues remained largely unaffected (Li et al., 2022). Furthermore, the ratio of synchronous fluorescence quenching (RSFQ = 1 − *F*/*F*_0_) for Trp was observed to be higher than that for Tyr residues (30.06% vs. 25.97% at 298.15 K), suggesting a greater contribution of Trp to fluorescence quenching in this study.

### 3.4 Docking conformation and detailed interactions of KAE with LF

**FIGURE 5:**
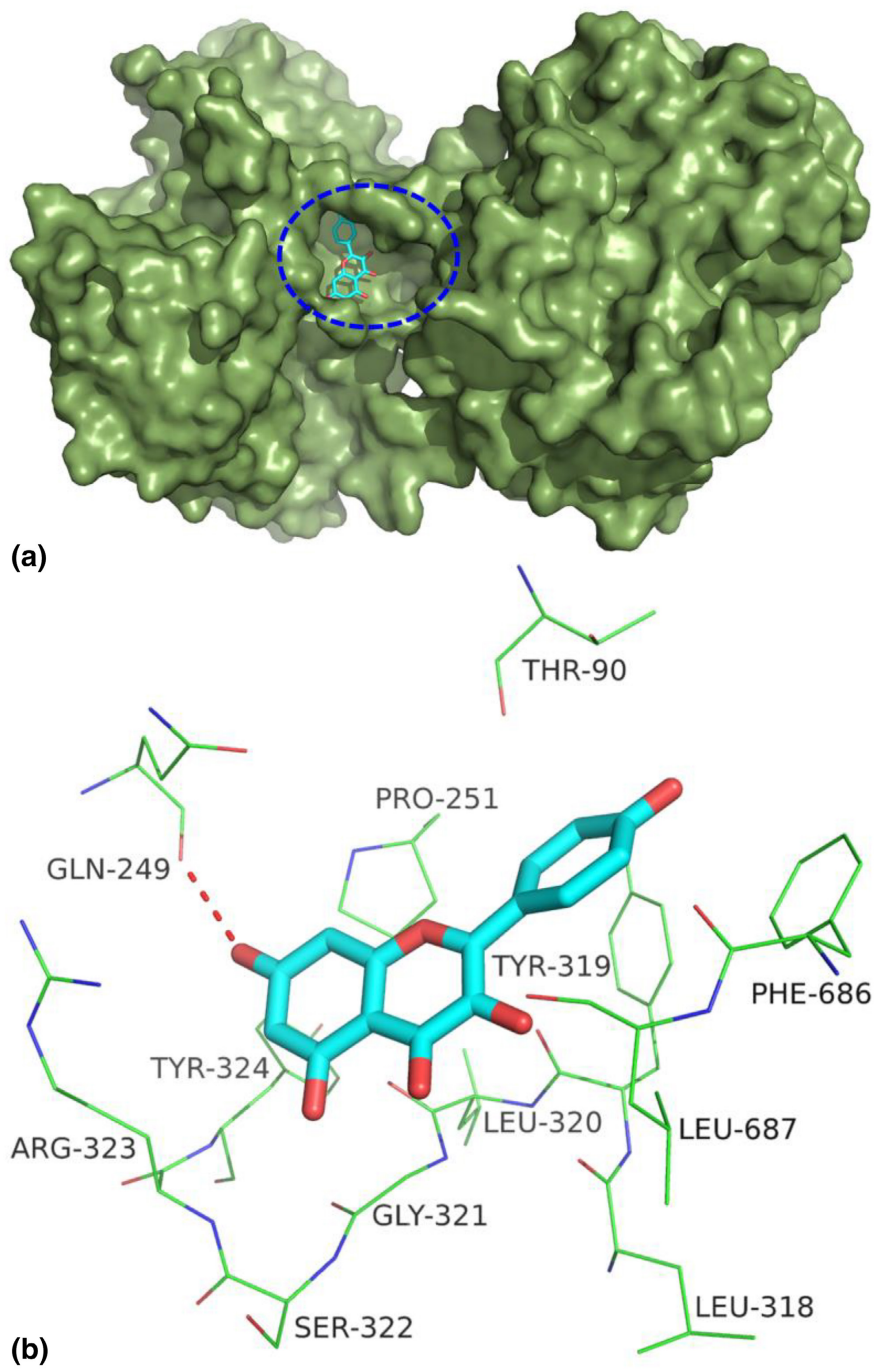
The docking conformation of kaempferol in the active pocket of lactoferrin (a) and their detailed interactions (b). The hydrogen bond is indicated by red dashed lines.

Molecular docking is an effective method to explain potential intermolecular interactions (Nayab et al., 2023). The result of molecular docking indicated that KAE can bind directly to the hydrophobic active pocket of LF, as shown in Figure 5a. In addition, it is observed in Figure 5b that KAE forms a hydrogen bond with the amino acid residue Gln249. It has been reported that this residue also formed hydrogen bond interactions with other natural phytochemicals such as tannic acid (Jing et al., 2021), which is consistent with the finding herein. The residues of LF that lie within 4 Å away from KAE can be seen in Figure 5b, among which, Pro251, Leu318, Leu320, Phe686, and Leu687 formed hydrophobic interactions with KAE. Collectively, these findings provide evidence that the interaction between LF and KAE is influenced significantly by both hydrogen bonding and hydrophobic interactions. The binding free energy of KAE with LF was −6.06 kcal/mol in this work, suggesting that KAE is a ligand of LF.

### 3.5 Binding stability of the LF–KAE complex

**FIGURE 6:**
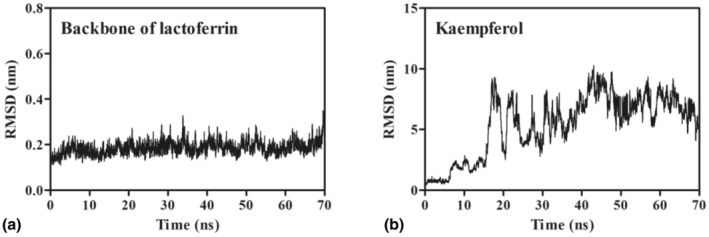
The results of a 70 ns molecular dynamics simulation for the backbone of lactoferrin (a) and kaempferol (b).

The molecular dynamics simulation lasting 70 ns was utilized to investigate the conjugation process of KAE with LF. As shown in Figure 6a, the backbone of LF appeared to have continuously high stability during the whole simulation process, with the average RMSD values of 0.19 ± 0.03 nm. It can be speculated that KAE causes a slight disturbance in the structure of LF. Unfortunately, KAE underwent a severe structural disturbance during the 70 ns simulation (Figure 6b). A possible explanation for this symptom is that KAE may bind to different active sites of LF.

### 3.6 Cytotoxicities of KAE and its complex with LF against HeLa cells

**FIGURE 7:**
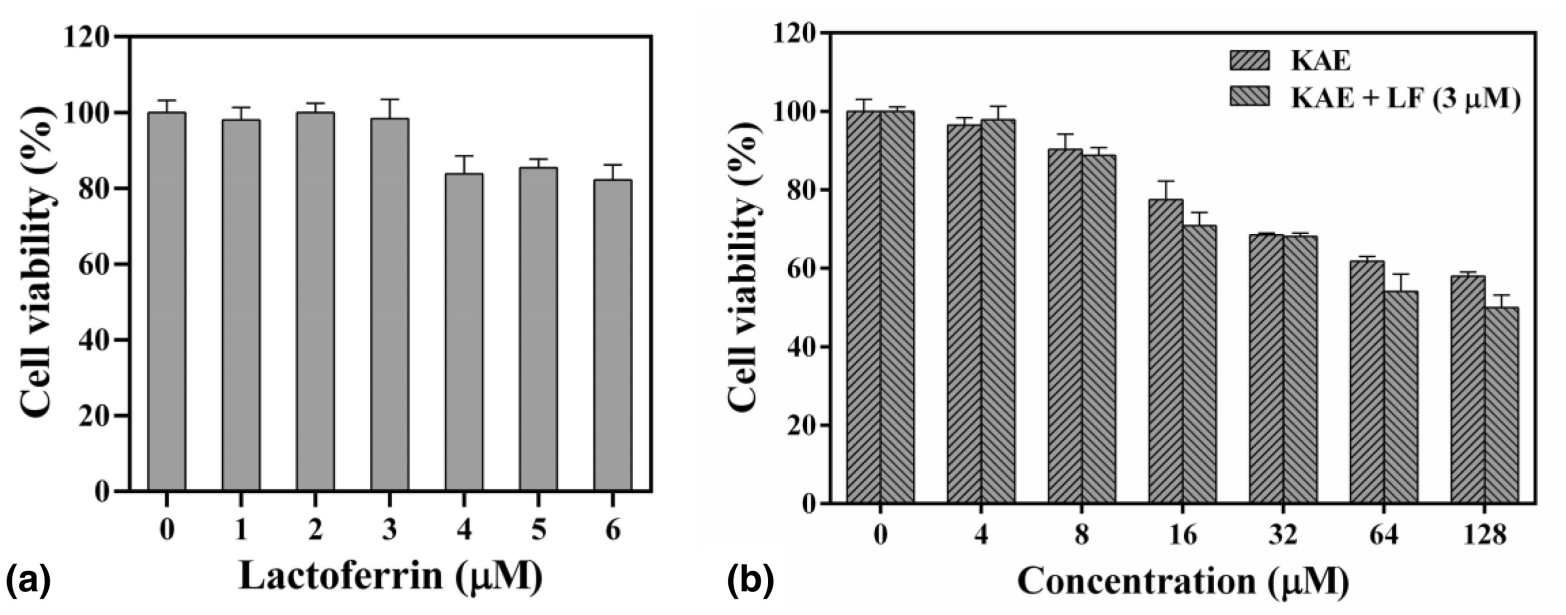
Cytotoxicities of lactoferrin, kaempferol, and their complexes against HeLa cells. (a) Lactoferrin. (b) Kaempferol (KAE) complexed with 3 μM lactoferrin (LF).

The MTT assay is a common method to evaluate the cell viability based on a determination of metabolic activity (Zaim et al., 2021). Briefly, succinate dehydrogenase within the mitochondria of living cells has the capability to reduce MTT into a water‐insoluble crystalline formazan, which is then deposited within the cells. Conversely, deceased cells are unable to complete this process, resulting in a generation of crystals that is directly proportional to the number of viable cells. The assessment of cell viability can be indirectly inferred by measuring the absorbance of formazan post‐dissolution (Cacciatore et al., 2016). MTT assays were employed to assess the inhibitory impact of LF, KAE, and their complexes on HeLa cells. Cell viability was not significantly affected when treated with LF concentrations below 3 μM (Figure 7a). Consequently, a fixed concentration of 3 μM LF was used for subsequent experiments. The cells were then treated with varying concentrations of KAE in combination with 3 μM LF, and cell viability was assessed (Figure 7b). The findings indicated that KAE was able to induce a significant increase in dose‐dependent cell death. Interestingly, when combined with LF, the inhibitory effect of KAE on cell viability was slightly enhanced compared to its individual treatment. Additionally, the inhibitory effects of different treatments were analyzed by calculating their respective IC_50_ values. The result revealed that KAE alone exhibited measurable inhibitory effects with an IC_50_ of 139.3 μM. In contrast, the combination of KAE and LF in the complex exhibited increased potency, with an IC_50_ value of 101.6 μM. These findings suggest a potential synergistic effect between KAE and LF in inhibiting HeLa cell growth and viability.

### 3.7 HeLa cell apoptosis induced by KAE and its complex with LF

**FIGURE 8:**
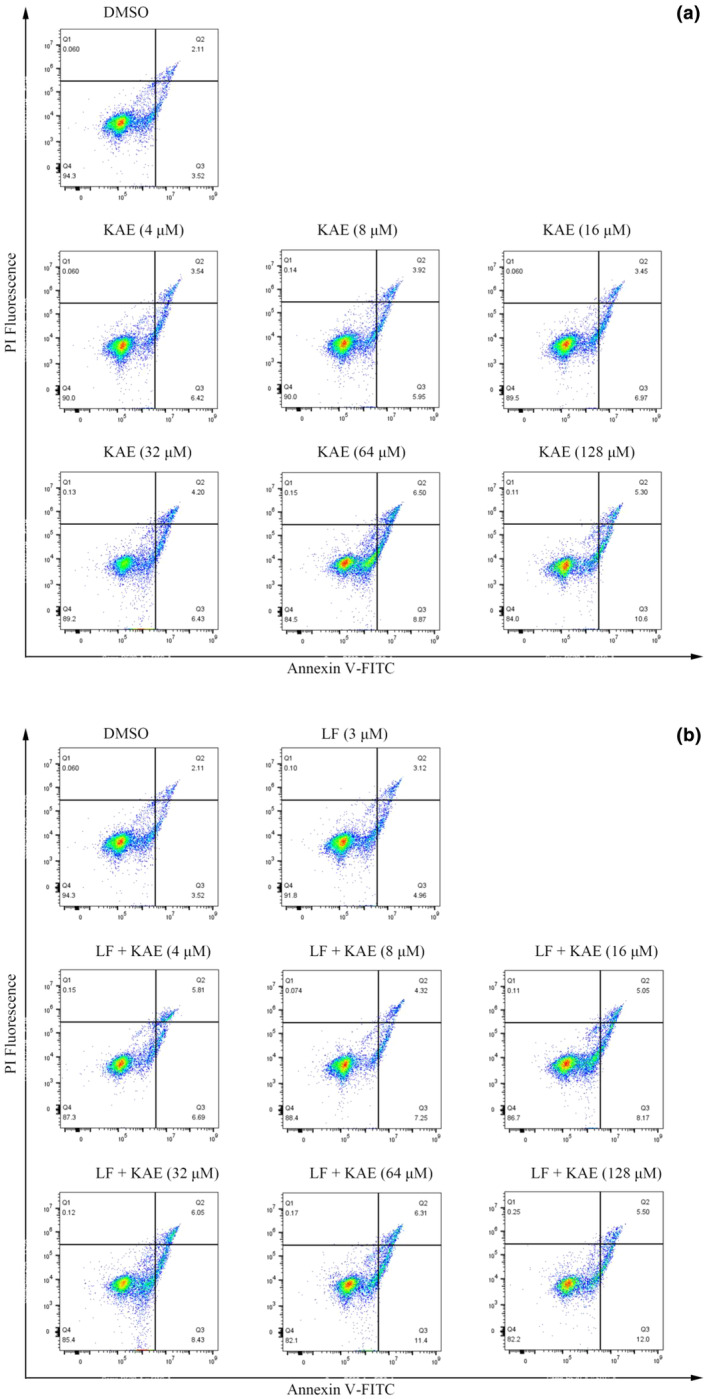
HeLa cell apoptosis induced by kaempferol (a) and its complex with lactoferrin (b). The DMSO‐treated group was used as a negative control.

Flow cytometry with Annexin V−FITC/PI double staining can be used to investigate cell apoptosis. During the early stage of apoptosis, phosphatidylserine undergoes a change in orientation on the cell membrane, allowing it to bind to Annexin V−FITC (Machala et al., 2022). As apoptosis progresses into the middle and late stages, PI is able to penetrate the cell membrane and stain the nucleus (Su et al., 2023). Therefore, the combination of Annexin V‐FITC and PI signals allows for the distinction of cells into four types. Annexin V−/PI−, Annexin V+/PI−, Annexin V+/PI+ and Annexin V−/PI+ represent viable cells, early apoptotic cells, late apoptotic cells, and necrotic cells, respectively (Easo & Mohanan, 2016). The induction of HeLa cell apoptosis by KAE and its complex with LF was further measured by flow cytometry. Unsurprisingly, the viability of cells was not affected by DMSO. However, the apoptotic cell rate increased with increasing concentrations of KAE (Figure 8a). In order to test whether LF enhanced the apoptosis‐inducing effect, HeLa cells were simultaneously treated with KAE and LF. As illustrated in Figure 8b, a concentration of 3 μM LF did not exhibit any cytotoxicity. However, the treatments of the LF–KAE complex resulted in a marked increase in the apoptosis rate. Notably, this increase was more significant compared to treatment with KAE alone. Even at a low concentration of 4 μM, a slight increase in apoptosis rate was observed after treatment with the complex. Moreover, the effects of the complex exhibited a similar trend to that of KAE treatment alone (Figure 8b). These findings indicate that KAE promotes the apoptosis of HeLa cells in a dose‐dependent manner, and LF has a synergistic effect in enhancing this apoptotic response.

## 4 CONCLUSIONS

This study has investigated the interaction between LF and KAE through the utilization of UV–visible absorption spectroscopy, fluorescence spectroscopy, molecular docking, and dynamics simulation techniques. Furthermore, the potential anticancer effects of the LF–KAE complex were assessed in HeLa cells through the MTT assay and flow cytometry. The findings from the spectra experiments indicated that KAE has the ability to form a complex with LF and effectively reduce the endogenous fluorescence of LF. The findings from the molecular docking and molecular dynamics simulations indicated that the binding between LF and KAE was influenced significantly by hydrophobic interactions and hydrogen bonding. A synergistic effect between KAE and LF in inhibiting the growth of HeLa cells was observed using the MTT assay and flow cytometry. The findings of this research can serve as a theoretical foundation for the application of LF and KAE in functional activities.

## AUTHOR CONTRIBUTIONS

**Peiyu Xue:** Writing – original draft (lead). **Hongmei Zhao:** Investigation (lead). **Xinyong You:** Writing – review and editing (equal). **Weiming Yue:** Writing – review and editing (equal).

## CONFLICT OF INTEREST STATEMENT

The authors declare that they have no known competing financial interests or personal relationships that could have appeared to influence the work reported in this paper.

## ETHICS STATEMENT

This study does not involve any human or animal testing.

## Data Availability

The data that support the findings of this study are available on request from the corresponding author.
